# Mental health effects of education

**DOI:** 10.1002/hec.4565

**Published:** 2022-07-07

**Authors:** Fjolla Kondirolli, Naveen Sunder

**Affiliations:** ^1^ University of Sussex Brighton UK; ^2^ Bentley University Waltham Massachusetts USA

**Keywords:** Africa, education, mental health, Zimbabwe

## Abstract

We analyze the role of education as a determinant of mental health. To do this, we leverage the age‐specific exposure to an educational reform as an instrument for years of education and find that the treated cohorts gained more education. This increase in education had an effect on mental health more than 2 decades later. An extra year of education led to a lower likelihood of reporting any symptoms related to depression (11.3%) and anxiety (9.8%). More educated people also suffered less severe symptoms – depression (6.1%) and anxiety (5.6%). These protective effects are higher among women and rural residents. The effects of education on mental well‐being that we document are potentially mediated through better physical health, improved health behavior and knowledge, and an increase in women's empowerment.

## 1 INTRODUCTION

Mental health accounts for around seven percent of the global disease burden and 19% of all disability years (James, 2018; Rehm & Shield, 2019). In addition to being a valuable end in itself, mental well‐being is critical because it is a key determinant of a number of socio‐economic outcomes such as premature mortality (Graham & Pinto, 2019) and lower life expectancy (Wahlbeck et al., 2011), and higher risk of other communicable and non‐communicable diseases (Hobkirk et al., 2015; Prince et al., 2007). In terms of economic outcomes, people with lower psychological well‐being have a higher likelihood of being unemployed (Frijters et al., 2014), earn lower wages (Graham et al., 2004), and are less productive (Oswald et al., 2015). This makes them vulnerable to economic shocks and more likely to live in poverty (Lund et al., 2011).

The negative effects of poor mental health are exacerbated in low‐ and middle‐income countries due to under‐treatment. Estimates suggest that under‐treatment is around 76–90% in low and middle‐income countries as opposed to 35–50% in developed countries (Patel et al., 2010). Mental health is stigmatized in low‐income countries – evidence from a variety of contexts including Ethiopia (Shibre et al., 2001), India (Koschorke et al., 2014), and Nigeria (Oshodi et al., 2014) demonstrates that individuals suffering from poor mental health fear being discriminated against and being ostracized by society, and consequently under‐report these issues and have a lower likelihood of seeking appropriate treatment. The under‐reporting of mental health issues, coupled with low investment in mental health infrastructure and diminished availability of human resources results in a higher treatment gap in these countries (Mascayano et al., 2015). The high prevalence and low rates of treatment of mental disorders in developing countries creates a large welfare loss ‐ Bloom et al. (2011) estimate that by the year 2030, mental disorders are expected to lead to a loss in economic output amounting to around 20% of the global GDP. This necessitates the need to study factors that can help improve mental health. We explore the role of one such factor – education.

In particular, we examine the effect of years of education on long‐run mental health of individuals. We do so in the context of Zimbabwe. Before 1980, the country was under British colonial rule, and there were several discriminatory policies that restricted educational attainment among Black Zimbabweans. For example, primary education (grades 1–7) was free and compulsory only for White students, while Black students had to pay fees and were not required to enroll. Black students also had to take a competitive exam (for a limited number of seats) to gain admission into secondary school, while their White counterparts received automatic progression. Post‐independence (in 1980) the new government focused their efforts on improving educational outcomes, and implemented three critical reforms ‐ (1) free and compulsory primary education for all, (2) automatic progression into secondary school, and (3) the relaxing of age restrictions related to entry into primary school. These reforms benefited Black children who were of primary school‐going age at the time. Since Zimbabwean kids started school when they turned six, those who were 13 years or younger in 1980 disproportionately benefited from these policies (treatment group). Since the reform also allowed for some over‐age students to enroll in school, some children who were 14 or 15 years of age in 1980 might have also experienced some educational gains (the partially treated group). However, older individuals (16 years or above at the time) were significantly less likely to experience any such benefits,^1^ and thus form our control group.

We utilize this age‐specific exposure to an educational reform in Zimbabwe in the 1980s as an instrumental variable (IV) for years of education to estimate the causal effect of schooling on later life mental health. We use an age cutoff of 15 years to define reform exposure – those who were 15 years and below in 1980 form the treated group, while those 16 years and older are considered as untreated. Our identification strategy rests on the assumption that individuals on either side of the age cutoff were, on average, conditionally indistinguishable, except for their exposure to this education policy, which led to higher levels of education among the treated. Our results indicate that the treated group eventually gained about three years of education and were 39% points more likely to attend secondary school. Our IV results suggest that this enhanced education led to better mental health later in life. We find significant effects of education on both the likelihood of having any adverse mental health related symptoms, and the severity of these symptoms. An extra year of schooling reduced the probability of reporting any symptoms related to depression (11.3%) or anxiety (9.8%) in adult life, and it also led to a decline in the severity of symptoms of both depression (6.1%), and anxiety (5.6%). Our findings suggest that the effects are larger among women and rural residents. We find evidence that improved physical health, better health‐related behavior and an increase in female empowerment might be some of the mechanisms through which education might have shaped mental health in the Zimbabwean context. We conduct a number of sensitivity analyses to show that our results are robust to various modifications in the empirical strategy, including levels at which the standard errors are clustered, and choice of bandwidth.

This paper contributes to two separate strands of literature. First, it adds to the growing evidence on the link between education and mental health.^2^ While some studies find a positive impact of education on mental well‐being (Chevalier & Feinstein, 2006; Courtin et al., 2019; Crespo et al., 2014; Dursun & Cesur, 2016; Jiang et al., 2020; Lager et al., 2017; Li & Sunder, 2022; Mazzonna, 2014; Wang, 2021), others document a negative effect (Avendano et al., 2020) or null effects (Begerow & Jürges, 2021; Böckerman et al., 2021). In addition to the mixed results of education on mental health, the bulk of this literature is based on developed countries, such as the UK, USA, Canada, Germany, and Sweden. We build on this literature by introducing novel evidence on the effects of education on mental health in Africa, a context where this relationship has not yet been explored. Second, this paper adds to the vast literature exploring the effects of education on health, which largely builds on the model of health accumlation proposed by Grossman (1972) model of health accumulation. A recent review article finds that the effect of education on health outcomes (such as mortality and obesity) and health behaviors (such as smoking) is highly context specific (Galama et al., 2018). This implies that evidence from one context might not be applicable to others, especially if they vary significantly. Moreover, it is unclear a priori whether the relationship between education and health extends to mental health. Our paper adds to the growing literature on the link between education and health by bringing forth evidence on mental health, a novel and understudied outcome.^3^

## 2 DATA AND KEY VARIABLES

The data used in this analysis comes from the World Health Survey (WHS). The WHS was conducted by the World Health Organization (WHO) across 70 countries between the years 2002 and 2004. The survey focused on topics related to health, and it aimed to generate detailed and synchronized information on population health and the state of health systems across the globe. We use data for Zimbabwe ‐ it was conducted in 2002 and sampled 4292 individuals across 4218 households. It is nationally representative, and the sample includes adults aged 18 years and above. Households were selected using a random, stratified sampling procedure, and one individual per household was selected for the interview. They also collected individual‐level information, including sociodemographic information, health state, health risk factors, chronic conditions, mortality, health care utilization, and social capital. We restrict our analysis to include people who were between the ages of zero and 30 in 1980, the year in which Zimbabwe implemented the education reform. This leaves us with a sample of 2604 individuals. Our analysis is based on the following self‐reported measures of mental health:

- *Depression Index*: This is based on the response to the following question: “Overall in the last 30 days, how much of a problem did you have with feeling sad, low or depressed?”. The responses are coded on a scale from one (“none”) to five (“extreme”).
- *Any Depression Symptoms*: This is a categorical variable that takes a value of one if the *Depression Index* takes a value greater than one.
- *Anxiety Index*: This is based on the response to the following question: “Overall in the last 30 days, how much of a problem did you have with worry or anxiety?”. The responses are coded on a scale from one (“none”) to five (“extreme”).
- *Any Anxiety Symptoms*: This is a categorical variable that takes a value of one if the *Anxiety Index* takes a value greater than one.
- *Mean Index*: This is a composite index created from the following measures: Depression Index, Anxiety Index, feeling depressed,^4^ lost interest^5^ and experiencing decreased energy.^6^ For each covariate, we create a standardized measure (with zero mean and a standard deviation of one) and then average across these standardized measures to get this index.

The summary statistics corresponding to these measures are reported in Table 1. The people in our sample have an average of around 8 years of education, and half of this sample has a secondary education. Around 70% of the individuals in our sample are male, and 60% live in rural areas. Nearly 40% of our sample reported suffering from some depression or anxiety‐related symptoms in the 30 days preceding the survey. The average severity of these reported symptoms in our sample are 1.8 for both depression and anxiety (measured on a scale ranging from one to five, where one is “none” and five is “extreme”).

**TABLE 1:** Descriptive statistics

	Full sample			Ages 0–30 in 1980		
	Obs.	Mean	S.D.	Obs.	Mean	S.D.
Years of education	4063	7.5	4.0	2583	8.0	3.8
Any secondary education (edu>7)	4063	0.5	0.5	2583	0.5	0.5
Age in 1980 (years)	4089	14.3	16.2	2604	11.8	8.6
Below 15 years in 1980	4100	0.6	0.5	2604	0.7	0.5
Male (=1)	4100	0.6	0.5	2604	0.7	0.5
Rural (=1)	4264	0.6	0.5	2588	0.6	0.5
Mean index (Z‐score)	4060	−0.0	0.8	2588	0.0	0.8
Depression index (1–5)	4048	1.8	1.1	2580	1.8	1.1
Any depression symptoms	4048	0.4	0.5	2580	0.4	0.5
Anxiety index (1–5)	4044	1.8	1.1	2578	1.8	1.1
Any anxiety symptoms	4044	0.4	0.5	2578	0.4	0.5

*Note*: This is based on data from the World Health Survey for Zimbabwe.

## 3 ZIMBABWE EDUCATION REFORM: BACKGROUND

Zimbabwe declared its independence from the British rule in 1980. Under colonial rule, the education policy in Zimbabwe was designed to favor White students at the expense of Black students. The Ministry of Education had separate departments for White and Black students, with widely varying budgets and policies, which discriminated against the Black population. The government spent 12 times more per primary school pupil (grades 1–7) and three times more per secondary school pupil (grades 8–11) in the “European” system as opposed to the “Black” system. Primary schooling was free and compulsory for White students, while Black students had to pay fees and enrollment was voluntary. There were limited number of seats in secondary school for Black schoolchildren, and allocation was based on a competitive exam. This was in contrast to White students who gained automatic progression into secondary schools (Dorsey, 1989; Nhundu, 1989, 1992).

After independence, the government implemented three key reforms aimed at equalizing educational opportunities for all – (1) government‐mandated free and compulsory primary schooling for all Zimbabweans, (2) automatic progression into secondary schools for everyone completing primary school (grades 1–7), and (3) the removal of age restrictions to allow over‐age children to enter school. To accommodate for the large demand for education, the government undertook a massive school building and reconstruction program. Between 1979 and 1981, the number of primary schools increased by 54%, while the number of secondary schools increased by 236% in the same period. The share of the budget allocated to education also increased from 11.6% in 1979–80 to 22.1% in 1980–81 (Nhundu, 1992).^7^ More details on the changes in number of schools, teachers, and education expenditure that accompanied this policy are provided in Appendix A.

**FIGURE 1:**
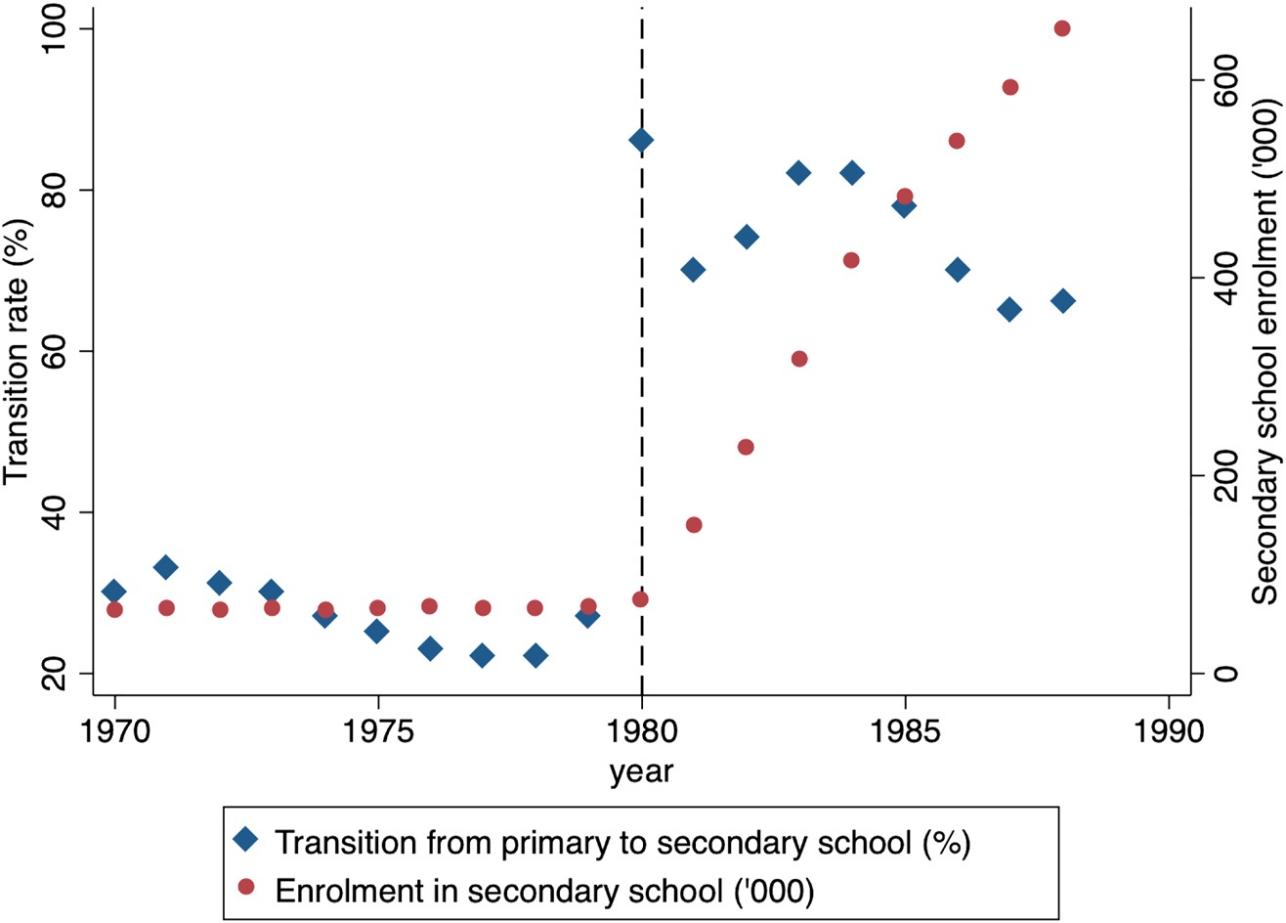
Trends in secondary school enrollment and transition for Zimbabwe. The estimates here are based on data from Riddell & Nyagura, 1991, which in turn are curated from the UN statistical yearbooks from 1970 to 1988. The transition rate is the percentage of students who graduated from grade seven (highest grade in primary school) who end up enrolling in grade eight (secondary school). Secondary school enrollment is measured in thousands of students.

Overall, this resulted in sizable increases in enrollment into primary school (from around 800,000 in 1979 to 2.2 million in 1986) and secondary school (from 66,215 in 1979 to 537,427 in 1986). The larger proportional increase was experienced in secondary school enrollment because of the high levels of discrimination experienced in transition from primary to secondary schools in the pre‐reform period. Therefore, the policies targeted toward a smoother transition from primary to secondary school (removal of the mandatory exam) led to huge educational benefits for the Black population. This is also illustrated by the following statistic – the percentage of seventh graders who joined secondary school increased from 20% in 1979 to about 78% in 1986. This significant rise in the post‐reform period is demonstrated in Figure 1 – the plot shows that there was a large jump in the percentage of students who transitioned from primary to secondary school starting in the year 1980. This figure also shows that total secondary school enrollment, which had largely stayed constant before 1980, increased steadily in this period (Figure 1).

Put together, these reforms disproportionately benefited Black children who were in primary school (grades 1–7) at the time (and would have progressed to secondary school in the ensuing years). School starting age in Zimbabwe was 6 years – therefore, children aged 13 years and below at the time of the reform would have gained from the passage of this policy. In our main analysis, we consider this as our fully treated group. Since the reform also allowed for some overage children to enroll in school, there is the possibility that some 14 and 15 year old children could have also benefited due to the reforms – we consider them as partially treated. Those who were 16 years and above in 1980 were considerably less likely to have benefited from this policy change and hence form our control group.

## 4 EMPIRICAL METHODOLOGY

To explore the relationship between education and mental health, one would estimate the following specification:

(1) $$Y_{i}=\alpha +\delta_{1}Education_{i}+\mu X_{i}+\epsilon_{i}$$

where *Y*_*i*_ is the mental health outcome for individual *i*, Education_i_ is the education level of individual *i*, and *X*_*i*_ includes gender, living in rural area, district and survey‐round fixed effects. OLS estimates of *δ*_1_, our coefficient of interest, would be biased because individuals with lower levels of education might also have other characteristics that could influence their mental health, such as lower income or worse physical health outcomes. To account for this bias, we use the exogenous shift in education caused by the Zimbabwean educational reform of 1980. As discussed earlier, this reform disproportionately increased access to education for Black children of primary school‐going age. Therefore, we create a categorical variable that divides our sample into a treated and a control group. Our treatment group consists of individuals who were 15 years or younger in 1980, and the control group consists of individuals who were 16 years or older at the same time. We implement a 2SLS‐IV framework, where the first stage equation uses the age‐specific exposure to the reform as an instrumental variable (IV) for years of education. In the second stage equation, we regress the outcome of interest on instrumented years of education. In this case, the equations of the first and second stage are as follows:

(2) $$Education_{i}=\delta_{1}+\delta_{2}Treated_{i}+f\left(Age80_{i}-15\right)+\sigma X_{i}+\nu_{i}$$

(3) $$Y_{i}=\alpha +\beta_{1}Education_{i}+f\left(Age80_{i}-15\right)+\gamma X_{i}+\epsilon_{i}$$

In Equation (3), *β*_1_ represents the causal impact of education on long‐run mental health outcomes. Education_i_ represents individual *i*'s education outcome, which in the main analysis will be years of education. In alternate specifications, we show that the results are robust to using a categorical variable for whether the individual had any secondary education (more than seventh grade). *Treated*_i_ is a categorical variable that takes a value of one for individuals who were 15 years of age or below in 1980, and zero otherwise. The covariate f(*Age80*_i_‐15) accounts for different functional forms of age. In the main specifications we include linear polynomials, while in robustness checks we control for higher‐order polynomials (such as quadratic). This controls for any cohort‐specific effect on individuals born in a given year.^8^

Standard errors are clustered at the level of the running variable, the age of the respondent in 1980. We control for gender, rural residence dummy, district fixed effects and temperature and rainfall shocks experienced by the individuals in their in‐utero period and infancy. The weather related controls are included to assuage concerns that these types of shocks experienced in childhood could be driving our findings (as shown by Adhvaryu et al., 2019, who find that temperature shocks in the in‐utero period increase depressive symptoms in adulthood in Africa). The data for rainfall and temperature is at the district level and comes from the Willmott and Matsura series (Matsuura & Willmott, 2018).^9^ In Appendix B, we further discuss the validity of this empirical strategy and present results from different checks, including McCrary density test (Figure A7) and falsification check related to effect on pre‐determined outcomes (Figure A8).

## 5 RESULTS

### 5.1 Impact of the 1980 reforms on educational attainment

**FIGURE 2:**
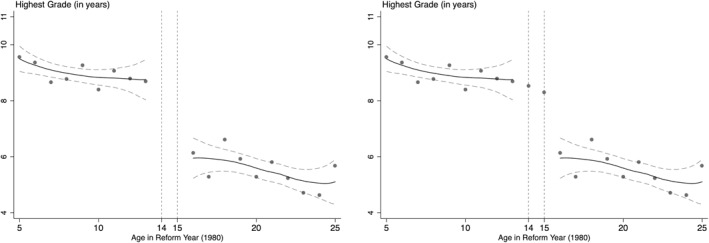
Effects of the Reform on Education (First Stage effects). Authors' estimate based on Word Health Survey data. The *y*‐axis represents the highest grade attained by individuals in our samples and the *x*‐axis represents the age in 1980 when the reform was implemented. All estimations include gender, living in rural area, fixed effects for region and survey rounds. Standard errors are clustered by the age of respondent in 1980.

We start by graphically examining the impact of the reform on educational attainment. To do this, we plot the highest grade attained (*y*‐axis) against age in the reform year (*x*‐axis). In Figure 2 we present graphs using the WHS data – the graph in the left panel demonstrates that years of education for the *fully treated group* (13 years and below) is considerably higher than that of the control group (16 years and above). The graph on the right in Figure 2 presents the same graph with the average years of education for 14 and 15 year olds (the partially treated group) included. The highest grade attained for the *partially treated* group is somewhere between the two aforementioned groups, confirming that some individuals of these cohorts benefited from the reforms. The same pattern is also observed using data from the Demographic and Health surveys^10^ and 2012 Zimbabwe census data (Figure A2). Since the reform particularly focused on the transition from primary to secondary school, it is instructive to see if there had been a significant rise in the rate of secondary schooling among the treated group. We present the graphs pertaining to the share of the sample with secondary schooling in Figure A1. The figure in the top panel suggests a discontinuous jump in the probability of attending secondary school among children aged 13 years and below, as compared to those who were 16 years or older at the time of the reform. Children who were 14 or 15 years of age again show a similar pattern as above (top panel, right graph). To demonstrate the robustness of the findings, we show that the effect of the reform is observed even when we use other datasets, the DHS (middle panel, Figure A1) and the 2012 census data (bottom panel, Figure A1).

We further probe the effects of this reform on education by conducting regression analysis. In particular, in Table 2, we present results corresponding to the first stage equation (Equation (2)). In our analysis sample, treated individuals had on an average 3.4 additional years of schooling as compared to the untreated (Table 2, column 1). This effect remains robust when restricting to smaller bandwidths around the age cutoff of 15 years (Table 2, columns 2 and 3). The impact estimate remains statistically significant when we restrict the sample to individuals between 6 and 23 years of age (3.8 years, *p*‐value < 0.001) and to those between 9 and 20 years of age (3.3 years, *p*‐value < 0.001). The impact is larger and remains statistically significant among individuals living in rural areas (Table 2, column 4). We present analogous results using any secondary education as an outcome in Table A3 ‐ the results follow a similar pattern to the findings above. The coefficient estimates of the impact of the reform on education presented here are larger in comparison to other studies in the same context. We posit that this is plausibly due to differences in the datasets used and the corresponding sample sizes in these analyses.

**TABLE 2:** First stage ‐ effect of reform on years of education

	(1)	(2)	(3)	(4)	(5)
Variables	Full sample	6–23 years	9–20 years	Rural	Urban
Below 15 years in 1980	3.38***	3.78***	3.34***	3.51***	3.16***
	(0.18)	(0.47)	(1.09)	(0.23)	(0.34)
Observations	2442	1455	958	1551	891
R‐squared	0.35	0.31	0.28	0.28	0.30
Mean ‐ full sample	7.92	7.96	6.20	6.97	9.57
Mean ‐ control	5.62	6.17	6.17	4.93	7.36
F‐stat (instrument)	348	63	9	227	89

*Note*: Results are based on data from World Health Survey from Zimbabwe. The full sample includes individuals who were between the ages of 0 and 30 in 1980. All specifications exclude those who were 14 and 15 years old in 1980, and control for categorical variables for living in a rural area, fixed effects for survey round, and region and linear age trends, and rainfall/temperature shock in the year of birth. Standard errors are clustered by the age of the respondent in 1980.

* Significant at the 10 percent level. ** Significant at the 5 percent level. *** Significant at the 1 percent level.

We conduct two separate checks to demonstrate the robustness of the first stage effects. First, we use other datasets to show that the effect of the policy on education that we observe in the WHS dataset is present in other nationally representative surveys ‐ the Demographic and Health Surveys (Table A1) and the 2012 Census data (Table A2). We find a positive and statistically significant effect of the policy reform on education across different sub‐samples in both datasets. Additionally, we also show that the first stage effects are largely insensitive to changes in the bandwidth used ‐ in this check we restrict the sample of analysis to between 5 and 12 years on either side of the cutoff age and find that the reform's effect on education is preserved in the WHS data (Figure A3), the DHS data (top panel of Figure A4) and the 2012 census data (bottom panel of Figure A4).

### 5.2 Impact of education on mental health

**FIGURE 3:**
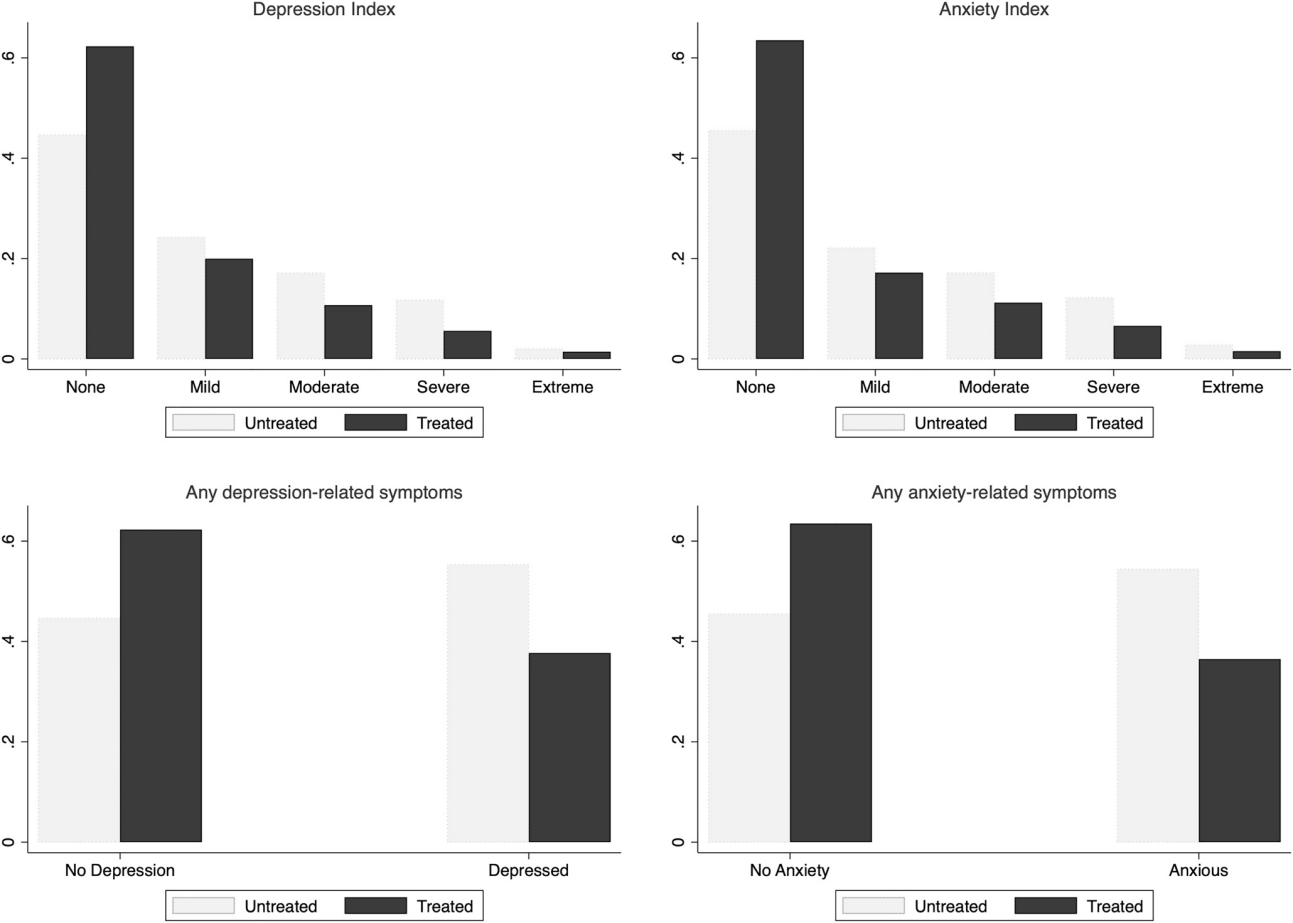
Values of mental health indices by treatment status. Based on Zimbabwe World Health Survey data. The figures plot the distribution of our measures of mental health: depression and anxiety indices, which measure the severity of symptoms related to depression and anxiety, in the top panels, and probability of having depression‐ or anxiety‐related symptoms in the bottom panels, by treatment status. Treated includes individuals aged 15 years and younger in 1980 and the untreated group consists of individuals aged 16 years and older in 1980.

Having established that the reform had a significant effect on educational outcomes, we explore whether this reform‐induced increase in education led to improved long‐run mental health. We first examine this using a graphical approach. In Figure 3, we plot the distribution of mental health indices by treatment status. This figure illustrates that the treated group are more likely to report any symptoms related to anxiety/depression and are less likely to experience more severe symptoms related to anxiety/depression. This points toward the fact that the reform had a positive effect on mental health.

**FIGURE 4:**
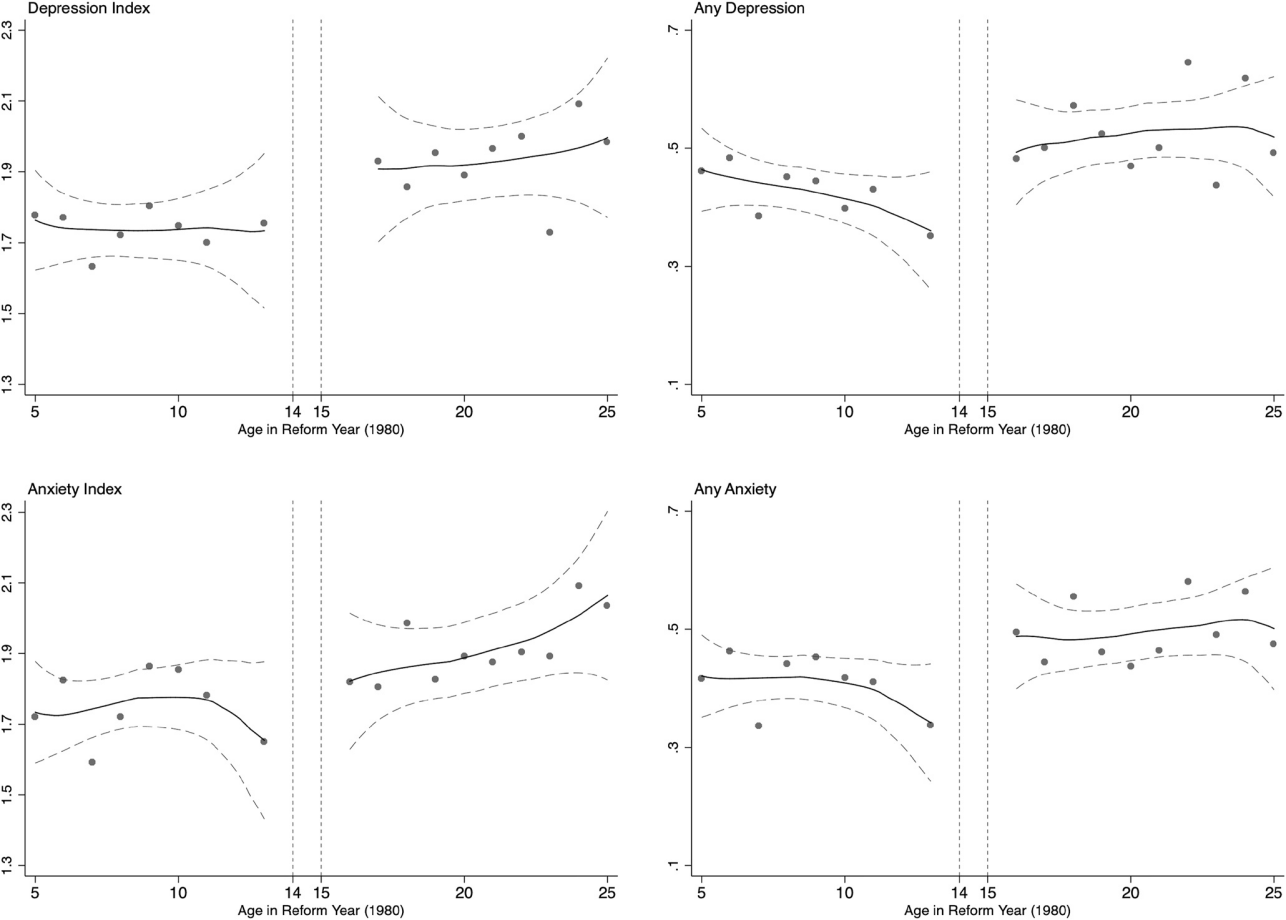
The impact of education on mental health. Based on author calculations using World Health Survey Zimbabwe. The figures plot different measures of mental health against the running variable (age in 1980). The upper panels represent depression index (left) which measures the severity of the symptoms and an indicator variable for having any depression‐related symptoms (right). The lower panels presents graphs for similar outcomes pertaining to anxiety‐related symptoms.

Further, in Figure 4, we present a plot of the impact of the reform on mental health. From this figure, we can conclude that the *fully treated* group (13 years and younger in 1980) is less likely to report symptoms related to anxiety or depression (Figure 4, right) and have lower intensity symptoms related to depression and anxiety (Figure 4, left).

As discussed in the empirical strategy section, the OLS specification (results in Table A4) will likely yield biased estimates of the impact of education on mental health due to the presence of other confounding factors such as income or physical health. Therefore, we conduct an IV analysis where years of education is instrumented using age‐based exposure to the Zimbabwean educational reform (based on Equations (2) and (3)). These results are presented in Table 3 and suggest that an increase in education leads to better mental health, a consistent pattern across different mental health measures. Education decreases the probability of having any depression related symptoms by 11.3% (Table 3, column 1) and those related to anxiety by 9.8% (Table 3, column 2).^11^ Additionally, education also reduces the severity of depressive symptoms by 6.1% (Table 3, column 3) and anxiety by 5.6% (Table 3, column 4). We also create a composite mental health index (combining the measures for depression and anxiety), where higher values indicate worse psychological health. We find that one more year of schooling leads to a decline in the composite mental health index by 0.06 standard deviations.

**TABLE 3:** IV regressions – education and mental health

	(1)	(2)	(3)	(4)	(5)
	Any depression	Any anxiety	Depression	Anxiety	Mean
Variables	Symptoms	Symptoms	Index	Index	Index
Years of education	−0.06***	−0.05***	−0.12***	−0.11***	−0.06***
	(0.01)	(0.01)	(0.02)	(0.02)	(0.01)
Observations	2429	2427	2429	2427	2436
Mean ‐ full sample	0.45	0.44	1.80	1.80	0.00
Mean ‐ control	0.53	0.51	1.96	1.95	0.09

*Note*: *Any Depression Symptoms* and *Any Anxiety Symptoms* are categorical variables measuring whether the respondent suffered from any depressive or anxiety related symptoms. *Depression Index* and *Anxiety index* are measured on a scale of 1–5, where 5 represents more severe symptoms. The sample includes individuals who were between the ages of 0 and 30 in 1980. All specifications exclude those who were 14 and 15 years old in 1980, and control for categorical variables for living in a rural area, fixed effects for survey round, region and linear age trends, and rainfall/temperature shock in the year of birth. The control mean here refers to the mean of the outcome variable among those who were 16–30 years of age at the time of the reform. Results are based on data from World Health Survey from Zimbabwe. Standard errors are clustered by the age of the respondent in 1980.

* Significant at 10 percent. ** Significant at 5 percent. *** Significant at 1 percent.

Comparing the coefficient estimates from the OLS specifications (Table A4) with those from the IV specifications (Table 3) shows that the OLS estimates are underestimates of the true causal effect. This is consistent with other studies estimating the causal impact of education on health (Agüero & Bharadwaj, 2014; Cutler & Lleras‐Muney, 2010) and mental health specifically (Dursun & Cesur, 2016). The OLS could underestimate the true causal effect for a number of reasons. First, omitted variables (e.g., social norms such as gender roles) which might be negatively correlated with education might lead to a downward bias on the observed coefficient. Second, measurement error in education could lead to attenuation bias which would reduce the magnitude of the education coefficient. Third, IV estimates LATE, which is different from the average treatment effect estimated by OLS. The LATE is estimated based on compliers – this group consists of individuals who, in the absence of the reform, would not have had as much schooling, not because of any differences in their ability levels but because of higher‐than‐average costs of schooling due to the oppressive policies aimed at creating educational bottlenecks for the Black population. These are likely to be the more disadvantaged among the Black population, who in turn are likely to have worse mental health. Hence it is possible that these individuals demonstrate a higher marginal effect of education on mental health ‐ thus leading to the IV estimates being higher than the OLS.

Our results are akin to other studies that have found a positive impact of education on mental health in other contexts. For example, Crespo et al., 2014 find that an additional year of schooling decreases the probability of suffering from depression by 6.5% points. As another example, Dursun & Cesur, 2016 find that an increase of 3 years of schooling in Turkey increased life satisfaction among women by 0.17 standard deviation. Studies that look at the impact of early‐life shocks/interventions also find similar results. Analyzing a compulsory schooling reform in China that increased schooling among beneficiaries, Li & Sunder, 2022 find that an increase of one year of schooling leads to a decrease of 26% in the likelihood of being depressed and a 10% decline in the severity of depressive symptoms.

We would like to note two potential caveats to our study. In comparison to administrative data, survey respondents are less likely to report mental health problems due to social stigma (Bharadwaj et al., 2017; Greene et al., 2015). Therefore it is possible that the prevalence of mental illness observed in our data is less than the actual rates. Another concern is that the reporting of mental health could be correlated with educational attainment. A priori it is unclear what the direction of the association would be – one could argue that more educated individuals are more likely to under report mental health issues due to social stigma, while on the other hand lesser educated individuals might face societal pressures that increase their likelihood of reporting a mental illness. However, due to lack of evidence directly linking education with self‐reports of mental health, we are unable to provide a direction to this bias.^12^

### 5.3 Heterogenous effects

Having established that an increase in education in childhood improves mental health later in life, we examine whether the relationship differs based on different socio‐economic characteristics.

**FIGURE 5:**
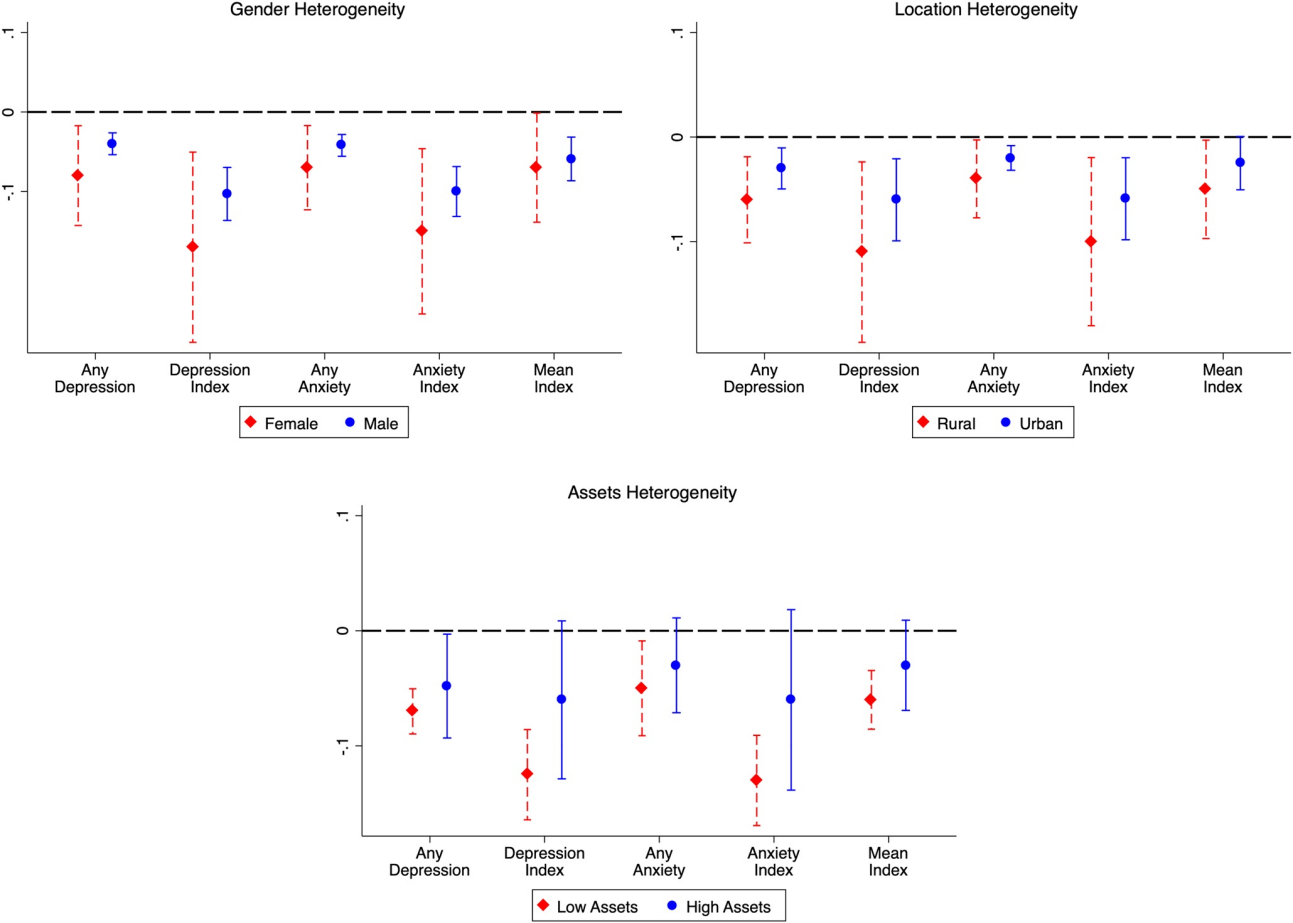
Heterogeneous impacts of education on mental health. The definitions of the mental health measures are the same as those used in Table 3. The sample includes women who were between the ages of 0 and 30 in 1980. All specifications exclude those who were 14 and 15 years old in 1980, and control for categorical variables for living in a rural area, fixed effects for survey round, region, linear age trends, and rainfall/temperature shock in the year of birth. Results are based on data from World Health Survey from Zimbabwe. Standard errors are clustered by the age of the respondent in 1980.

First, we look at the impact of education on mental health by gender. Enrollment data shows that the Zimbabwean reform had a larger effect on girls ‐ the number of girls who enrolled in secondary school increased by tenfold between 1980 and 1985 (Chikuhwa, 2008). Therefore, we might expect that this increased educational effect might translate into larger positive impacts on women's mental health. Results in Figure 5 provide suggestive evidence supporting this ‐ education has a larger effect on mental health for women. One more year of education among women reduces the probability of having depression (14.8%) and anxiety (13%) related symptoms, while it also lowers the and the severity of these symptoms – depression (8.2%) and anxiety (7.1%). These effect sizes are larger than the overall effects, but the difference is not statistically significant. However, these results provide suggestive evidence of higher mental health gains among women. This gender‐differentiated impact is critical for two reasons. First, women have a higher prevalence of mood‐related disorders than men, possibly due to biological differences as well as lower self‐esteem, the experience of gender‐based violence, and gender discrimination (Boyd et al., 2015; Riecher‐Rössler, 2017). Our results suggest that education might mitigate some of these effects. Second, these mental health gains confer some intergenerational (indirect) benefits as well – studies have demonstrated that improved maternal mental health is associated with higher educational attainment, future household income, lower probability of criminal convictions (Johnston et al., 2013), and better health outcomes (Le & Nguyen, 2018). Since a majority of women in our sample are in the reproductive age range, improved mental health can not only improve their own well‐being, but also have strong implications for the long‐term human capital accumulation of the next generation as well.

Next, we assess the impact of education on mental health among individuals living in rural areas. This is motivated by prior studies have found higher effects of educational reforms in rural areas (Erten & Keskin, 2018; Li & Sunder, 2022). In our case, the treated group in rural areas had 3.5 more years of education (Table 2, column 4). This large increase in education in rural areas does translate into a higher positive impact on their mental health. Rural residents were seven p.p. and four p.p. less likely to report any symptoms related to depression and anxiety respectively in adulthood (Figure 5).

Finally, we examine whether individuals belonging to households with lower wealth experience any heterogeneous effects. In low‐ and middle‐income countries, low income is strongly associated with mental disorders (Rehm & Shield, 2019). Additionally, in developing country contexts, like Zimbabwe, the lower access to mental health resources and the inability of the poor to afford any available resources further adds to the problem. In this study, we measure assets using a composite asset index (created using principal component analysis^13^) and define a household having low assets if it lies in or below the 25th percentile of the composite asset index distribution. Results in Figure 5 show that the impact of one additional year of schooling on mental health is indeed larger for this sub‐sample.

### 5.4 Robustness checks

We conduct several robustness checks to examine the sensitivity of our main empirical findings.

#### 5.4.1 Bandwidth

We check whether our findings regarding the impact of education on mental health are robust to the use of different bandwidths around the age cutoff of 15 years. We replicate our main analysis using different bandwidths around the cutoff age – these bandwidths vary between 5 and 12 years, in 1‐year increments. The coefficient estimates from this exercise are presented in Figure A5 – they indicate that the effects of education on mental health are always negative (more education leads to better mental health) and largely remain statistically significant. This suggests that our main findings are largely insensitive to changes in bandwidth.

#### 5.4.2 Polynomial function

In our main specification, we use a linear polynomial to control for the running variable, the age of the individual in 1980 minus 15. As a robustness check, we control for the running variable using a quadratic polynomial. The results presented in Figure A6 suggest that the impact of an extra year of education on all our measures of poor mental health remain negative and statistically significant.

#### 5.4.3 Clustering of standard errors

We examine how sensitive our results are to clustering of the standard errors at different levels. In the main specification, standard errors are clustered by the age of the individual. But, there might be a high degree of correlation between the outcomes of people who live close to each other (in the same region), and hence including the region in the clustering may be desirable. Therefore, in Figure A6 we present results using two‐way clustering of the standard errors at the region‐age level. The results remain statistically significant.

#### 5.4.4 Including partially treated individuals

As mentioned earlier, our main analysis excludes individuals who were 14 and 15 year old in 1980 (since they were partially treated the reform). Therefore, as a robustness check, we include them in the treated sample and re‐run our analysis. We find that the results remain similar in both size and statistical significance (Figure A6).

#### 5.4.5 Changes in empirical strategy

Finally, we employ a slightly different empirical strategy than the one that is used in the main analysis (IV strategy). Here, we divide the treatment group into two separate categories – those who were completely treated, which includes individuals who were aged seven or below in 1980 (likely started primary school in/after 1980), and those who were partially treated, referring to people who were between 8 and 14 years of age in 1980 (likely to have already started primary school before the policy was implemented). The intuition behind creating these two groups is that individuals who were exposed to the policy throughout their primary schooling (completely treated) are likely to experience more benefits than those who were only benefited from it for a part of their primary schooling years (part treated). Results in Table A6 show that one more year of education among completely treated individuals led to a larger decrease in depression and anxiety symptoms, as well as the probability of reporting depression or anxiety symptoms compared to those who were only part treated, which is in line with our prior expectations.

### 5.5 Mechanisms

Further, we probe the potential mechanisms that might drive the observed effects of education on mental health in this context. Using various strands of medical, economic, and psychological literature, we explore several possible channels through which the mental health effects that we observe could have been mediated.

#### 5.5.1 Physical health

Across a variety of contexts, it has been shown that physical health is strongly positively associated with mental health (Ohrnberger et al., 2017; Sabia & Rees, 2015; Willage, 2018). We examine whether improvements in education among the treated group in Zimbabwe led to improved physical health later in life. We measure physical health using the following indicators – self‐rated health (=1 if reported good health), whether the person had difficulty working in the past 30 days, whether they experienced any pain or discomfort in the last 30 days, and a categorical variable for whether their BMI was in the normal range (between 18.5 and 25). The results from this analysis are presented in Table 4 – they indicate that higher education led to better outcomes across all these indicators. More educated people reported better health (5 p.p.), experienced fewer difficulties/pain and had a five p.p. higher likelihood of having a BMI in the normal range. Additionally, they were seven p.p. and six p.p. less likely to report difficulty in working or any pain or discomfort, respectively. This implies that the Zimbabwean reform led to improved physical health outcomes, which in turn could be an important factor in driving the observed effects on mental health.^14^

**TABLE 4:** Mechanisms – Physical health

	(1)	(2)	(3)	(4)
	Self Rated	Diffulty	Any pain	Normal
Variables	Health	Working	Or discomfort	BMI
Years of education	0.05***	−0.07***	−0.06***	0.05***
	(0.01)	(0.01)	(0.01)	(0.01)
Observations	2442	2389	2442	1545
Mean ‐ full sample	0.19	0.39	0.50	0.59
Mean ‐ control	0.11	0.5	0.59	0.55

*Note*: The sample consists of individuals who were between 0 and 30 years of age in 1980 (excluding 14 and 15 year old). The control mean here refers to the mean of the outcome variable among those who were 16–30 years of age at the time of the reform. Standard errors are clustered by the age of the respondent in 1980.

* Significant at 10 percent. ** Significant at 5 percent. *** Significant at 1 percent.

*Source*: World Health Survey Zimbabwe.

#### 5.5.2 Health knowledge and behaviors

Past studies have demonstrated that improved health‐related practices (such as not smoking, lower alcohol consumption, and a healthy diet) are positively associated with mental well‐being (Buttery et al., 2015; Parletta et al., 2016). Therefore, we consider the following possibility: did educational gains among the treated cohorts also lead them to have improved health behaviors later in life, which in turn could have positively affected psychological health? In this analysis, we proxy health behaviors and knowledge using three different outcomes ‐ smoking/alcohol related behavior, HIV knowledge,^15^ and knowledge/usage of various forms of contraception. With respect to smoking and alcohol consumption, we find that more educated individuals are less likely to be smoking (3 p.p.) or consuming alcohol (4 p.p.). The results in Table 5 also indicate a positive effect of schooling on enhanced knowledge of HIV among treated women – we find that women with higher education have more comprehensive knowledge about HIV (2 p.p.). Additionally, an increase in 1 year of education makes women five p.p. more likely to know about contraception^16^ and increases their likelihood of using contraception by three p.p. (Table 5, columns 4–5). Overall, this suggests that improved health‐related behaviors might be an important mediator of mental health gains.

**TABLE 5:** Mechanisms – Health knowledge and behaviors

	(1)	(2)	(3)	(4)	(5)
	Any	Any	Comprehensive	Knows all	Ever used
Variables	Alcohol	Smoking	HIV knowledge	Contraception	Contraception
Years of education	−0.04***	−0.03***	0.02***	0.05***	0.03***
	(0.01)	(0.01)	(0.01)	(0.01)	(0.01)
Observations	2416	2416	13,224	2856	13,224
R‐squared	0.19	0.15	0.31	0.01	0.56
Mean ‐ full sample	0.21	0.11	0.32	0.43	0.60
Mean ‐ control	0.28	0.16	0.22	0.38	0.69

*Note*: The sample consists of individuals who were between 0 and 30 years of age in 1980 (excluding 14 and 15 year old). The control mean here refers to the mean of the outcome variable among those who were 16–30 years of age at the time of the reform. Standard errors are clustered by the age of the respondent in 1980.

* Significant at 10 percent. ** Significant at 5 percent. *** Significant at 1 percent.

*Source*: DHS Zimbabwe 1994, 1999, 2005.

#### 5.5.3 Women's empowerment

There is growing evidence of a robust negative association between the prevalence of gender‐based violence and various measures of psychological challenges, including suicides, depression, post‐traumatic stress, and eating disorders (Grose et al., 2019). Using information from multiple rounds of the Zimbabwe DHS, we examine whether women's empowerment could have been a potential channel for the observed mental health effects. In particular, we explore the following three different dimensions of empowerment:

- *Decision‐making power:* This is measured using responses to questions on whether the women were involved in making decisions within the household (either by themselves or jointly with their husband).^17^ We largely find no statistically significant effect of increased education on any of these measures of women's decision‐making (Table 6, Column 1).
- *Prevalence of IPV:* Our findings suggest that women in the treated group have a 22 percent lower likelihood (relative to control mean) of reporting being the victim of sexual violence by their partners in the past 12 months. They are also less likely to have ever experienced physical and sexual, and emotional violence by their current partner (17 percent) (Table 6, Columns 2 and 3).
- *Women's employment:* Women's employment increases her decision‐making within the household, and hence is considered an important proxy of empowerment (Anderberg et al., 2016; Majlesi, 2016). In our case, we find that more educated women are more likely to be employed in salaried work outside the household (3 p.p.) (Table 6, Column 4).
  Put together, these results point toward the role of increased empowerment among women, in the form of education‐induced reductions in IPV prevalence and increases in employment, as one of the potential mediators of the mental health benefits that we document.

**TABLE 6:** Mechanisms – Women's empowerment

	(1)	(2)	(3)	(4)
	Number of	Sexual	Physical, sexual	Employed
Variables	Decisions	Violence	& emotional violence	
Years of education	−0.00	−0.02**	−0.01*	0.03***
	(0.02)	(0.01)	(0.01)	(0.01)
Observations	6197	6269	6269	13,223
Mean ‐ full sample	1.72	0.10	0.06	0.48
Mean ‐ control	1.72	0.09	0.06	0.51

*Note*: *Sexual violence* is an indicator that takes the value of one if the woman experienced sexual violence from her partner in the last 12 months, and zero otherwise. *Sexual & physical & emotional violence* is an indicator that takes a value of one if the woman has ever experienced sexual, physical, and emotional violence from her partner, and zero otherwise. The sample consists of individuals who were between 0 and 30 years of age in 1980 (excluding 14 and 15 year old). The control mean here refers to the mean of the outcome variable among those who were 16–30 years of age at the time of the reform. Standard errors are clustered by the age of the respondent in 1980.

* Significant at 10 percent. ** Significant at 5 percent. *** Significant at 1 percent.

*Source*: DHS Zimbabwe 1994, 1999, 2005.

## 6 DISCUSSION AND CONCLUSION

Mental health is a growing priority among policymakers, as indicated by its inclusion in the United Nations' Sustainable Goals (SDG). Despite the acknowledgment of its importance, investment in mental health has remained low, especially in Africa.^18^ This has led to a shortfall in the availability of medicine, infrastructure, and health workers, which has resulted in large‐scale undertreatment of mental illness, the costs of which will become increasingly higher as the continent is expected to double its population in the next 3 decades (Sankoh et al., 2018). To the extent possible, our study examines whether, in contexts with limited health infrastructure, other complementary investments in childhood, such as education, could help decrease the burden of poor mental health in adulthood.

We study whether there is a causal link between education and mental health. We do so by leveraging the exogenous shift in education caused by a policy intervention in Zimbabwe. This reform removed significant barriers to education that Black schoolchildren in Zimbabwe faced and was effective in improving educational outcomes among the target population. Using nationally representative survey data and IV‐2SLS methodology, we find that an additional year of education reduces the probability of reporting any symptoms related to depression (11.3%) and anxiety (9.8%). Also, increased education has a dampening effect on the severity of symptoms related to both depression (6.1%) and anxiety (5.6%). Our results also indicate that the impact of education on mental health is larger for women and rural residents. In terms of mechanisms, we find that physical health, health knowledge (and behaviors) and women's empowerment might be crucial drivers of our findings.

This evidence on the protective effects of education on mental health is especially significant when viewed in conjunction with the mixed results demonstrated by other direct (and indirect) mental health‐enhancing interventions in similar contexts. For example, a review of the evidence on the relationship between poverty and depression and anxiety found that the average impact of anti‐poverty programs is a decrease in 0.094 SD in common mental health disorders such as depression and anxiety (0.138 SD for multi‐faceted anti‐poverty programs and 0.067 for cash transfer programs) (Ridley et al., 2020). These interventions are one‐off, potentially resource‐intensive, and affect only a small share of the population. In contrast, in this study, we show that large‐scale policy reform such as an expansion of education has a large and persistent long‐term impact on mental health, complementing similar evidence from other countries (Chevalier & Feinstein, 2006; Courtin et al., 2019; Crespo et al., 2014; Dursun & Cesur, 2016; Jiang et al., 2020; Lager et al., 2017; Mazzonna, 2014; Wang, 2021). These benefits become even more significant (and cost‐effective) since these education policies were not specifically designed to target these health outcomes and are spillover effects.

Our paper adds to the growing evidence on the efficacy of education in improving health outcomes in general (Galama et al., 2018) and to the understanding of the impact of interventions during adolescence on economic and social outcomes in adulthood (Cunha et al., 2010; Heckman, 2007). Our findings motivate future research on the impact of large‐scale policies in developing countries on understudied outcomes such as mental health, even if the interventions themselves were not directly targeted toward it.

## CONFLICT OF INTEREST

The authors have no conflict of interest to declare.

## Supporting information

Supporting Information S1

## Data Availability

World Health Survey data is available online at https://apps.who.int/healthinfo/systems/surveydata/index.php/catalog/whs/about. Demographic and Health Surveys data is available online at https://dhsprogram.com/. Zimbabwe census data is available online at https://www.zimstat.co.zw/.

## ENDNOTES

^1^
Using multiple data sources (including census data), we demonstrate in our analysis that those who were 16 years or older had a much lower likelihood of experiencing educational gains due to this policy. This pattern has also been confirmed by other studies of the same policy intervention (Agüero & Ramachandran, 2020; Grépin & Bharadwaj, 2015).

^2^
Broadly, economic research on mental health has largely focused on developed countries, and has examined different determinants of mental well‐being, such as income (Baird et al., 2013; Haushofer & Shapiro, 2016; Kahneman & Deaton, 2010; Kahneman & Krueger, 2006; Stevenson & Wolfers, 2013), poverty (Haushofer & Fehr, 2014; Lund et al., 2011; Tsaneva & Balakrishnan, 2019), environment (Zhang et al., 2017), socioeconomic status (Lorant et al., 2003; Stewart‐Brown et al., 2015), crime (Bor et al., 2018; Cornaglia et al., 2014; Dustmann & Fasani, 2016; Metcalfe et al., 2011), early‐life circumstances (Adhvaryu et al., 2019; Dinkelman, 2017; Persson & Rossin‐Slater, 2018), career choices (Bertrand, 2013), residential status (Katz et al., 2001), and migration (Scheffel & Zhang, 2019).

^3^
Other papers that have looked at the effect of education on health (and related) outcomes in the African context include child mortality (Grépin & Bharadwaj, 2015; Keats, 2018), fertility (Osili & Long, 2008), HIV prevalence (Duflo et al., 2015), and teenage pregnancy (Baird et al., 2013).

^4^
Takes a value of one if the response to the following question is “Yes” (and zero otherwise): “During the last 12 months, have you had a period lasting several days when you felt sad, empty or depressed?”

^5^
Takes a value of one if the response to the following question is “Yes” (and zero otherwise): “During the last 12 months, have you had a period lasting several days when you lost interest in most things you usually enjoy such as hobbies, personal relationships or work?”

^6^
Takes a value of one if the response to the following question is “Yes” (and zero otherwise): “During the last 12 months, have you had a period lasting several days when you have been feeling your energy decreased or that you are tired all the time?”

^7^
The demand for education could not be met with a proportional increase in the supply of teachers in years immediately after 1980, which possibly led to the government hiring untrained and under‐qualified teachers (according to some sources the share of untrained teachers was as high as 43 percent in 1987 (Nhundu, 1992)). To increase the supply of trained teachers, the University of Zimbabwe offered a 2‐year part‐time course for teachers who were already working in secondary schools and the government introduced the Zimbabwe Integrated National Teacher Education Course, that consisted of an one‐term teaching course followed by a three and half years of teaching in primary (and subsequently secondary) schools.

^8^
Any differences between the treatment and control cohorts due to factors not controlled for in our specification would be a concern if they were to differentially affect those that are just below and those just above the cutoff age – which we think this is unlikely to be the case. Having said that, we note this as a caveat of this analysis. Additionally, we do not know of any other policies or reforms implemented concurrently in Zimbabwe that was targeted in the same manner as this education reform.

^9^
Rainfall and temperature shocks are defined using the following formula: log(average over a given 12 month period) minus log(long term mean).

^10^
We use the 1994, 1999, and 2005 rounds of the Demographic and Health surveys in Zimbabwe.

^11^
The effect sizes are relative to control group mean. Since the reform targeted entry into secondary school, in an alternate specification we use a categorical variable for having any secondary education as the main independent variable of interest (instead of years of education). As expected, having any secondary education has a large positive impact on mental health (estimates in Table A5).
