# Supplementary material for: Mental health effects of education
Source: Health Econ. 2022 Jul 7;31(Suppl 2):22–39. doi: 10.1002/hec.4565 (PMC9796491; doi:10.1002/hec.4565)
Supplement: Supplementary file 1 — Supporting Information S1 [file HEC-31-22-s001.pdf]

# Appendix Tables & Figures

Table A.1: **First Stage - Effect of Reform on Years of Education (Using DHS data)**

| VARIABLES | (1)<br>Full Sample | (2)<br>6-23 years | (3)<br>9-20 years | (4)<br>Rural | (5)<br>Urban |
|---|---|---|---|---|---|
| Below 15 years in 1980 | 2.08*** | 1.70*** | 1.20*** | 2.53*** | 1.05*** |
| | (0.14) | (0.19) | (0.26) | (0.14) | (0.24) |
| Observations | 17,839 | 10,126 | 6,206 | 12,122 | 5,717 |
| R-squared | 0.322 | 0.289 | 0.268 | 0.266 | 0.159 |
| Mean-Y | 7.4 | 7.2 | 7 | 6.6 | 9.2 |
| F-stat (instrument) | 235 | 82 | 22 | 310 | 20 |

Notes: Results are based on data from the Zimbabwe Demographic and Health Survey (1994, 1999, 2005). The full sample includes individuals who were between the ages of 0 and 30 in 1980. All specifications exclude those who were 14 and 15 years old in 1980, and control for categorical variables for living in a rural area, fixed effects for survey round, and region and linear age trends. Standard errors are clustered by the age of the respondent in 1980. * Significant at 10 percent. ** Significant at 5 percent. *** Significant at 1 percent.

Table A.2: **First Stage - Effect of Reform on Years of Education (Using 2012 Census)**

| VARIABLES | (1)<br>Full Sample | (2)<br>6-23 years | (3)<br>9-20 years | (4)<br>Rural | (5)<br>Urban |
|---|---|---|---|---|---|
| Below 15 years in 1980 | 2.14*** | 1.63*** | 0.98*** | 2.27*** | 1.80*** |
| | (0.13) | (0.16) | (0.18) | (0.12) | (0.20) |
| Observations | 72,825 | 37,154 | 21,682 | 49,762 | 23,063 |
| R-squared | 0.404 | 0.352 | 0.329 | 0.341 | 0.205 |
| Mean-Y | 7.6 | 7.4 | 8.3 | 6.6 | 9.8 |
| F-stat (instrument) | 266 | 101 | 30 | 333 | 82 |

Notes: Results are based on data from the 2012 Zimbabwe Census data. The full sample includes individuals who were between the ages of 0 and 30 in 1980. All specifications exclude those who were 14 and 15 years old in 1980, and control for categorical variables for living in a rural area, fixed effects for survey round, and region and linear age trends. Standard errors are clustered by the age of the respondent in 1980. * Significant at 10 percent. ** Significant at 5 percent. *** Significant at 1 percent.

Table A.3: **First Stage - Effect of Reform on Any Secondary Education**

| VARIABLES | (1) Full Sample | (2) 6-23 years | (3) 9-20 years | (4) Rural | (5) Urban |
|---|---|---|---|---|---|
| Below 15 years in 1980 | 0.28*** | 0.23*** | 0.20*** | 0.39*** | 0.22*** |
| | (0.01) | (0.02) | (0.03) | (0.01) | (0.03) |
| Observations | 17,852 | 10,132 | 6,211 | 12,130 | 5,722 |
| R-squared | 0.267 | 0.266 | 0.254 | 0.142 | 0.185 |
| Mean-Y | 7.5 | 7.2 | 7 | 6.6 | 9 |
| F-stat (instrument) | 378 | 122 | 44 | 2885 | 52 |

Notes: Results are based on data from World Health Survey from Zimbabwe. The full sample includes individuals who were between the ages of 0 and 30 in 1980. All specifications exclude those who were 14 and 15 years old in 1980, and control for categorical variables for living in a rural area, fixed effects for survey round, and region and linear age trends. Standard errors are clustered by the age of the respondent in 1980. * Significant at 10 percent. ** Significant at 5 percent. *** Significant at 1 percent.

Table A.4: **OLS Regressions – Education and Mental Health**

| VARIABLES | (1) Any Depression Symptoms | (2) Any Anxiety Symptoms | (3) Depression Index | (4) Anxiety Index | (5) Mean Index |
|---|---|---|---|---|---|
| Years of Education | -0.01** | -0.01* | -0.02** | -0.02** | -0.01* |
| | (0.004) | (0.004) | (0.007) | (0.007) | (0.005) |
| Observations | 2,429 | 2,427 | 2,429 | 2,427 | 2,436 |
| R-squared | 0.122 | 0.109 | 0.117 | 0.129 | 0.135 |
| Mean - Full Sample | .45 | .44 | 1.8 | 1.8 | 0 |
| Mean - Control | .53 | .51 | 1.96 | 1.95 | .09 |

Notes: Results are based on data from World Health Survey from Zimbabwe. *Any Depression* and *Any Anxiety* are categorical variables measuring whether the respondent suffered from any depressive or anxiety related symptoms. *Depression Index* and *Anxiety index* are measured on a scale of 1 to 5, where 5 represents more severe symptoms. The sample includes individuals who were between the ages of 0 and 30 in 1980. The control mean here refers to the mean of the outcome variable among those who were 16 to 30 years of age at the time of the reform. All specifications exclude those who were 14 and 15 years old in 1980, and control for categorical variables for living in a rural area, fixed effects for survey round, and region and linear age trends, and rainfall/temperature shock in the year of birth. Standard errors are clustered by the age of the respondent in 1980. * Significant at 10 percent. ** Significant at 5 percent. *** Significant at 1 percent.

Table A.5: **IV Regressions – Education (Any Secondary Education) and Mental Health**

| VARIABLES | (1)<br>Any Depression<br>Symptoms | (2)<br>Any Anxiety<br>Symptoms | (3)<br>Depression<br>Index | (4)<br>Anxiety<br>Index | (5)<br>Mean<br>Index |
|---|---|---|---|---|---|
| Any Secondary Edu (=1) | -0.50*** | -0.42*** | -1.05*** | -0.99*** | -0.55*** |
| | (0.098) | (0.081) | (0.185) | (0.171) | (0.114) |
| Observations | 2,429 | 2,427 | 2,429 | 2,427 | 2,436 |
| R-squared | -0.118 | -0.082 | -0.124 | -0.103 | -0.064 |
| Mean - Full Sample | .45 | .44 | 1.8 | 1.8 | 0 |
| Mean - Control | .53 | .51 | 1.96 | 1.95 | .09 |

*Note:* Notes: Results are based on data from World Health Survey from Zimbabwe. *Any Depression* and *Any Anxiety* are categorical variables measuring whether the respondent suffered from any depressive or anxiety related symptoms. *Depression Index* and *Anxiety index* are measured on a scale of 1 to 5, where 5 represents more severe symptoms. The sample includes individuals between the ages of 0 and 30 in 1980.The control mean here refers to the mean of the outcome variable among those who were 16 to 30 years of age at the time of the reform. All specifications exclude those who were 14 and 15 years old in 1980, and control for categorical variables for living in a rural area, fixed effects for survey round, and region and linear age trends, and rainfall/temperature shock in the year of birth. Standard errors are clustered by the age of the respondent in 1980. * Significant at 10 percent. ** Significant at 5 percent. *** Significant at 1 percent.

Table A.6: **Robustness Check – Different Methodology**

| VARIABLES | (1)<br>Any Depression<br>Symptoms | (2)<br>Any Anxiety<br>Symptoms | (3)<br>Depression<br>Index | (4)<br>Anxiety<br>Index | (5)<br>Mean<br>Index |
|---|---|---|---|---|---|
| Completely Treated | -0.24*** | -0.22*** | -0.52*** | -0.57*** | -0.27** |
| | (0.06) | (0.05) | (0.10) | (0.11) | (0.11) |
| Part Treated | -0.20*** | -0.17*** | -0.42*** | -0.41*** | -0.22*** |
| | (0.03) | (0.02) | (0.05) | (0.05) | (0.04) |
| Observations | 2,439 | 2,437 | 2,439 | 2,437 | 2,447 |
| R-squared | 0.135 | 0.120 | 0.132 | 0.142 | 0.144 |
| Mean - Full Sample | .45 | .44 | 1.8 | 1.8 | 0 |
| Mean - Control | .53 | .51 | 1.96 | 1.95 | .09 |

Notes: Results are based on data from World Health Survey from Zimbabwe. *Any Depression* and *Any Anxiety* are categorical variables measuring whether the respondent suffered from any depressive or anxiety related symptoms. *Depression Index* and *Anxiety index* are measured on a scale of 1 to 5, where 5 represents more severe symptoms. Partially treated refers to people who were between 8 and 14 years of age in 1980, while fully treated refers to people who were seven or below in 1980. The sample includes individuals between the ages of 0 and 30 in 1980. All specifications exclude those who were 14 and 15 years old in 1980, and control for categorical variables for living in a rural area, fixed effects for survey round, and region and linear age trends, and rainfall/temperature shock in the year of birth. Standard errors are clustered by the age of the respondent in 1980. * Significant at 10 percent. ** Significant at 5 percent. *** Significant at 1 percent.

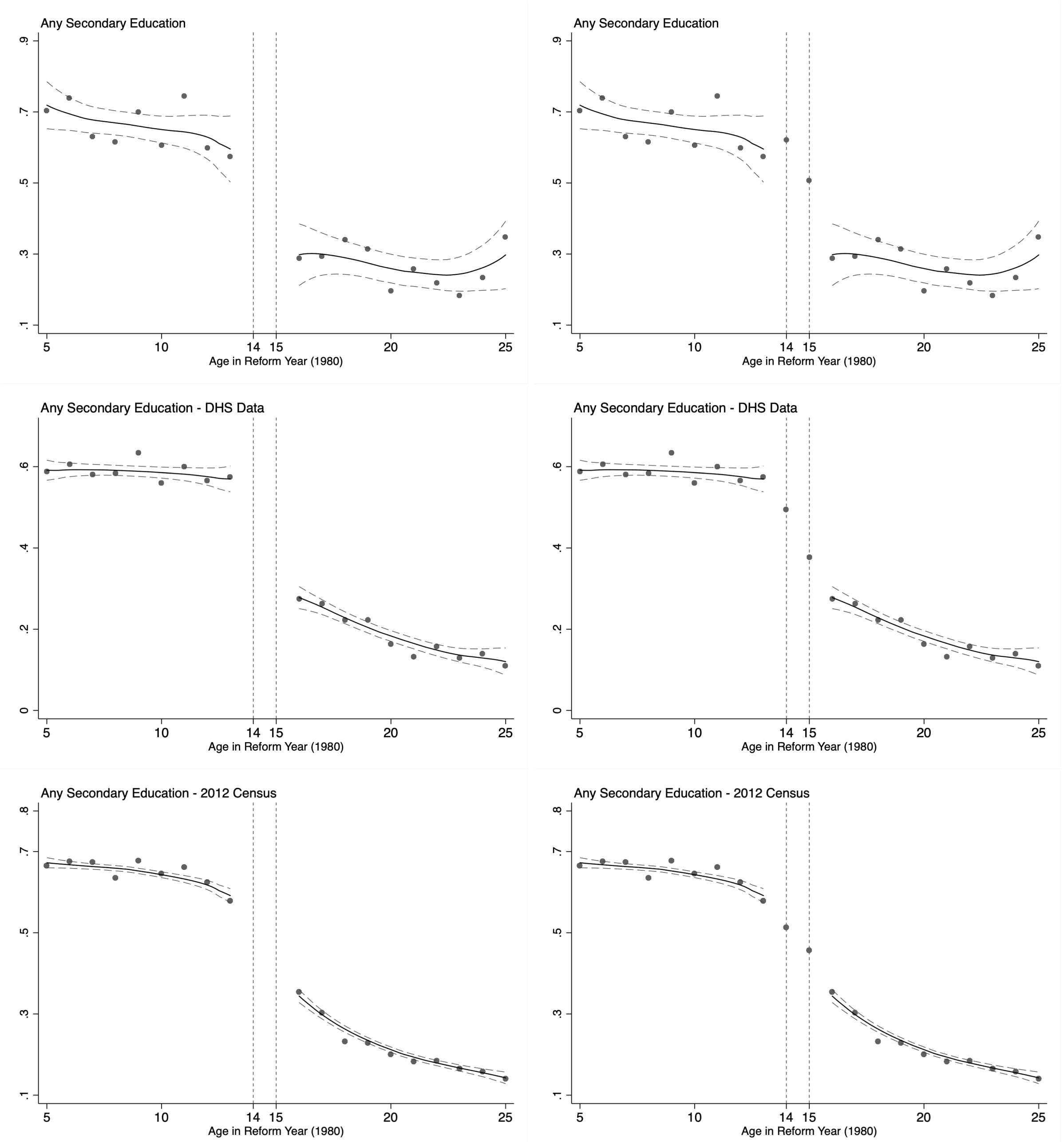


Figure A.1: **Effects of the Reform on any secondary education (First Stage effects)**

Notes: Upper panel is based on Word Health Survey data, the middle panel is based on combined DHS data, and the lower panel is based on census data. Individuals aged 14 and 15 years old in 1980 are excluded in the left panels and included in the right panels. The y-axis represents the share of the sample with a secondary education and the x-axis represents the age in 1980 when the reform was implemented. All specifications control for categorical variables for living in a rural area, fixed effects for survey round, and region and linear age trends. Standard errors are clustered by the age of the respondent in 1980.

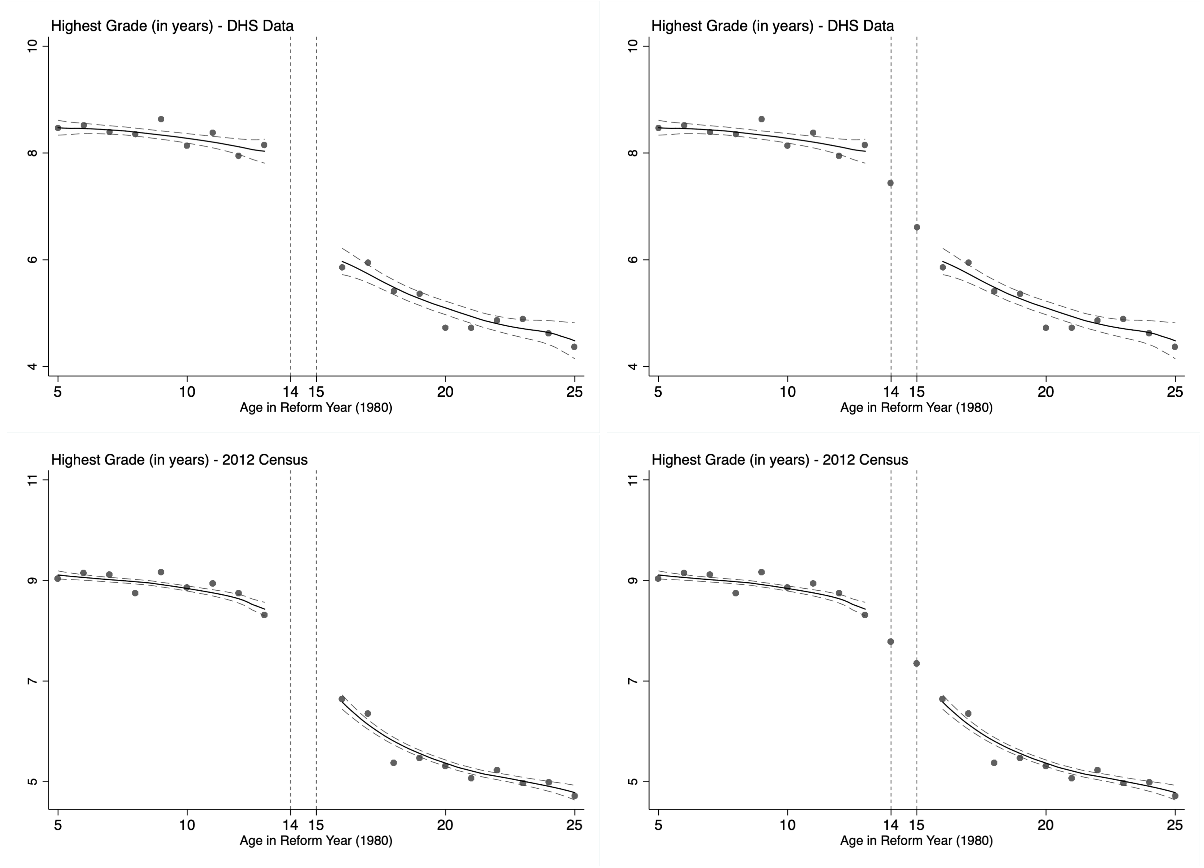


Figure A.2: **Effects of the Reform on Education (First Stage effects)**

Notes: Upper panel is based on DHS data, while the lower panel is based on census data. Individuals aged 14 and 15 years old in 1980 are excluded in the left panels and included in the right panels. The y-axis represents the highest grade attained by individuals in our samples and the x-axis represents the age in 1980 when the reform was implemented. All estimations include gender, living in rural area, fixed effects for region and survey rounds. Standard errors are clustered by the age of respondent in 1980. The vertical line in each graph represents the cutoff point.

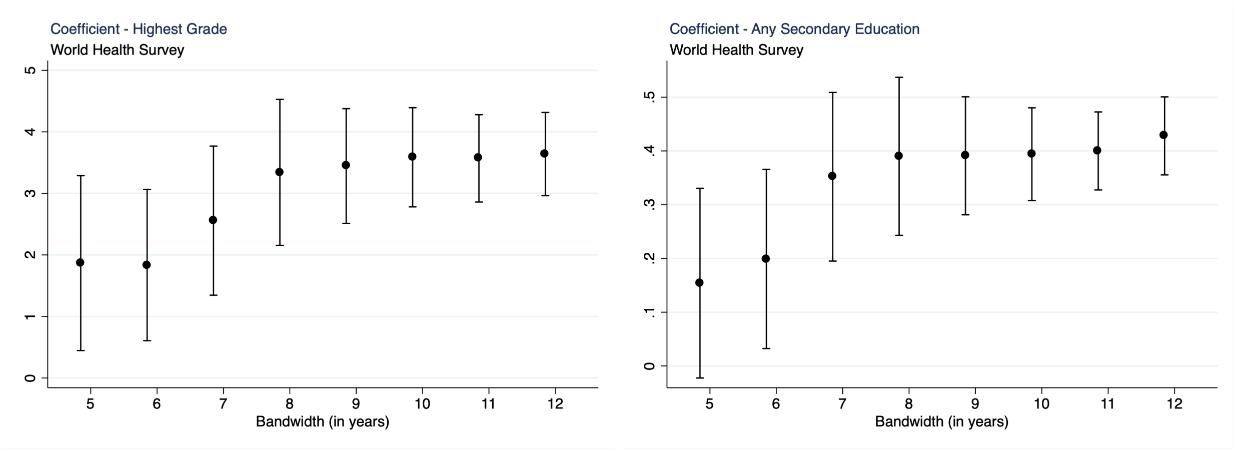


Figure A.3: **First Stage effects - for different bandwidths around the cutoff**

Notes: Based on Word Health Survey data. Each point represents an estimate of the impact of the reform on years of education (left panel) and any secondary education (right panel). The estimations are calculated for bandwidths from 5 years to 12 years, with one year increments. All estimations control for gender, living in rural area, and district fixed effects. Standard errors are clustered by the age of the respondent in 1980.

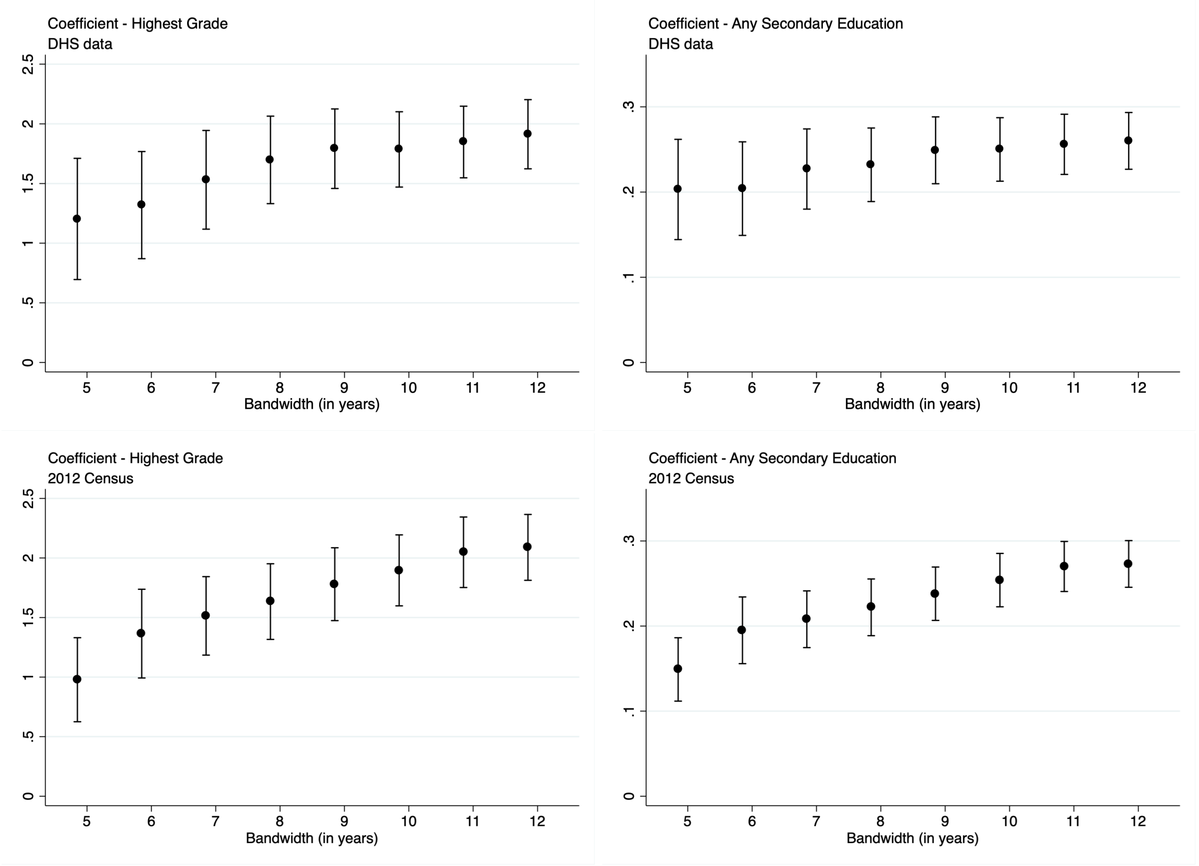


Figure A.4: **First Stage effects - for different bandwidths around the cutoff**

Notes: Upper panel is based on combined DHS data, and the lower panel is based on census data. Each point represents an estimate of the impact of the reform on years of education (left panels) and having any secondary education (right panels). The estimations are calculated for bandwidths from 5 years to 12 years, with one year increments. All estimations control for gender, living in rural area, fixed effects for region and survey rounds. Standard errors are clustered by the age of the respondent in 1980.

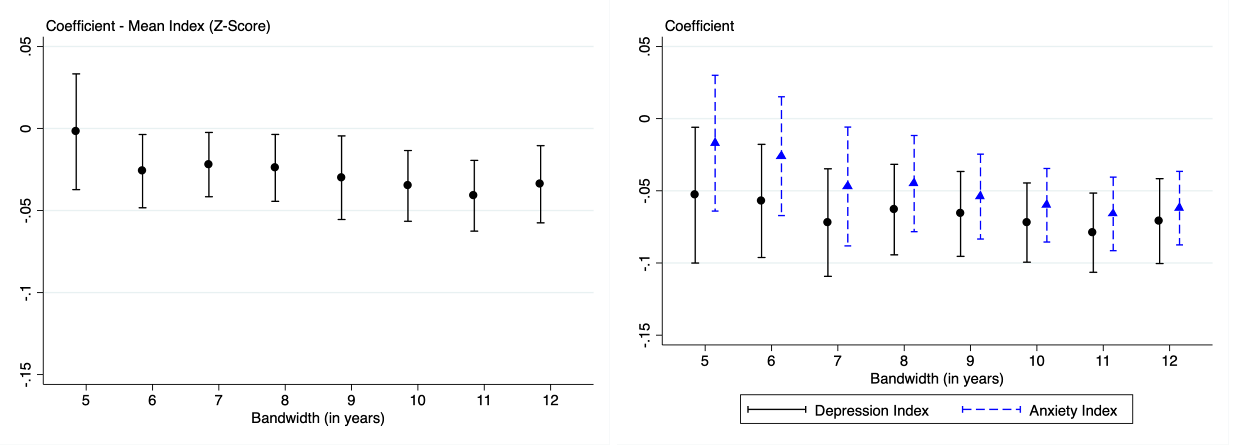


Figure A.5: **RD impact estimates – for different bandwidths around the cutoff**

Notes: Based on Zimbabwe World Health Survey data. Each point represents an estimate of the impact of education on our standardized measure of mental health on the left panel, and depression index and anxiety index on the right panel. The estimations are calculated for bandwidths starting with 5 years to 12 years, with one year increments.The controls for all specifications include categorical variables for living in a rural area, fixed effects for survey round, and region and linear age trends, and rainfall/temperature shock in the year of birth. The standard errors are clustered by the age of the respondent in 1980.

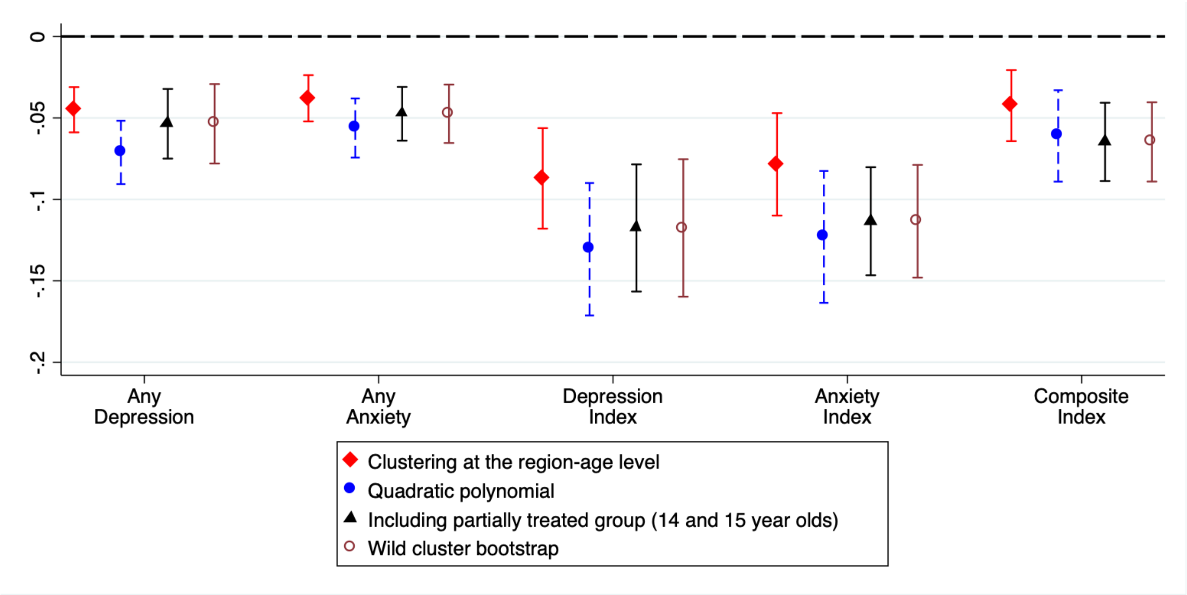


Figure A.6: **Robustness checks**

Notes: Based on Zimbabwe World Health Survey data.
See data section for a detailed description of the outcomes of interest.
*Clustering at the region-age level*: The sample includes individuals between the ages of 0 and 30 in 1980. All specifications exclude those who were 14 and 15 years old in 1980, and control for categorical variables for living in a rural area, fixed effects for district, linear age trends, and rainfall/temperature shock in the year of birth. Results are based on data from World Health Survey from Zimbabwe. Standard errors are clustered by district of residence and the age of the respondent in 1980.
*Including partially treated group (14 and 15 years old)*: The sample includes individuals between the ages of 0 and 30 in 1980. All specifications control for categorical variables for living in a rural area, fixed effects for district, linear age trends, and rainfall/temperature shock in the year of birth. Results are based on data from World Health Survey from Zimbabwe. Standard errors are clustered by the age of the respondent in 1980.
*Quadratic polynomial*: The sample includes individuals were between the ages of zero and 30 in 1980. All specifications exclude those who were 14 and 15 years old in 1980, and control for categorical variables for living in a rural area, fixed effects for district, quadratic age trends, and rainfall/temperature shock in the year of birth. Results are based on data from World Health Survey from Zimbabwe. Standard errors are clustered by the age of the respondent in 1980.

# Appendix A

## 1980 Education Reform in Zimbabwe

Below, we examine changes pertaining to three different aspects of school infrastructure (schools, teachers and education budget):

**Number of primary and secondary schools:**

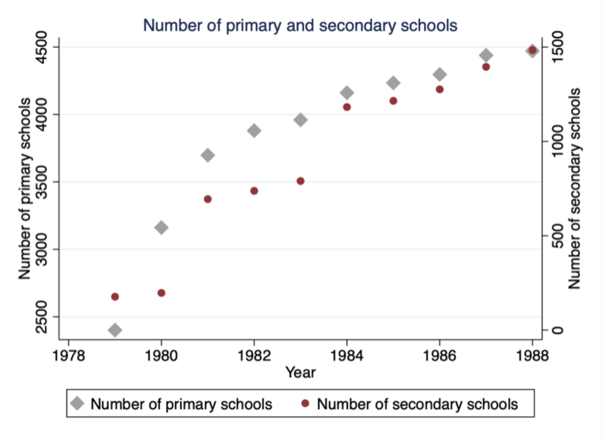


Between 1979 and 1981, the number of primary schools increased by 54 percent, while the number of secondary schools increased by 236 percent in the same period (Nhundu, 1992). This represents a substantial increase in the supply of school infrastructure in the immediate aftermath of the announcement of the policy.

**Availability of skilled teachers:**

We could not obtain reliable statistics on the actual number of teachers in primary and secondary schools in this period. Therefore, we use the number of students enrolled in teacher colleges (institutions that provides training to future school teachers) as a proxy for the availability of new teachers after the reforms were implemented.

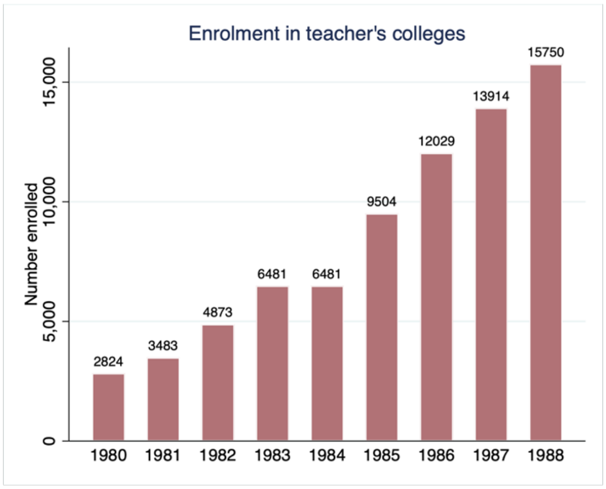


Between 1980 and 1982, the number of students enrolled in teacher colleges increased from 2,824 to 4,873, which translates to an increase of around 73 percent (data source – Nhundu, 1992). This implies that there was a rise in the number of potential teachers available in the country – but this increase would take effect with a lag as it would take at least two to four years for these people to join the teaching workforce. In the interim, there were a lot of untrained teachers hired, and they formed around 43 percent of the teaching workforce (Nhundu, 1992).

**Budget expenditure on education (at the national level):**

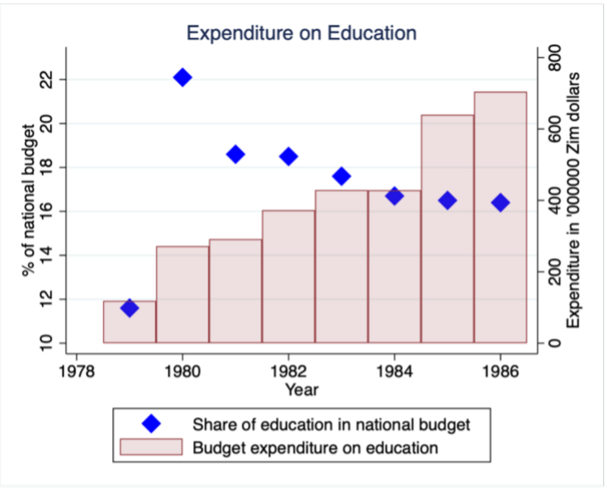


Between 1979 and 1981, the education budget (in absolute value) rose by 145 percent. Thereafter, there was a steady increase in the education budget every year, as demonstrated by the adjoining figure. However, the share of the budget spent on education increased from 11.6 percent in 1979 to 22.1 percent in 1980. This was representative of the intentions of the government – on gaining independence in 1980 there was a dramatic shift in focus towards education. Following that period (between 1981 and 1986), the relative expenditure on education hovered around 16-18 percent (data source – Nhundu, 1989).

Overall, these findings suggest that there was a significant and sudden increase in education infrastructure and investment in 1980-81, followed by a gradual increase in the following years. This supports our empirical strategy that leverages this shift in education policy in 1980 (and beyond) to create an instrumental variable for years of education – we consider all cohorts that would enter secondary school after 1980 (age <= 15 years in 1980) to have benefitted from the policy.

# Appendix B

## Validity of the empirical strategy

Our IV-2SLS empirical setup is akin to a regression discontinuity design (RD). A critical assumption of the RD design is that the observations above and below the treatment cutoff should be observationally similar (Hahn et al., 2001). This implies that the treatment is as good as random around the cutoff, and it enables the use of the average outcome of those below the cutoff as the counterfactual for those who are above the threshold (Lee and Lemieux, 2010). This assumption is not directly testable as we cannot observe the counterfactual. Instead, we conduct several indirect tests that suggest the credibility of our RD design.

For the RD design to be valid, it is critical that individuals are unable to manipulate their treatment status by systematically positioning themselves on either side of the RD cutoff. If individuals could manipulate their position relative to the cutoff, then it would lead to non-random treatment assignment around the cutoff, which would invalidate causal interpretation of our results. To alleviate this concern, we conduct two checks. First, we visually examine the distribution of the running variable around the RD cutoff. The graph, presented in the top left panel of Figure A.7, shows that there are no dramatic jumps in density around the cutoff. This suggests the absence of any systematic differential sorting of individuals around the RD cutoff. Second, we conduct two statistical tests to add strength to this observation. We check for differences in density across the RD cutoff by conducting the McCrary density test on the running variable (as described in McCrary, 2008). The intuition behind this test is that if there is systematic manipulation in the forcing variable, it would be observed via a heaping of observations around the cutoff. The graph (top right panel of Figure A.7) shows no such discontinuous jumps in the distribution of the running variable around the RD cutoff. The null hypothesis of the associated McCrary density test is that there is no heaping around the RD cutoff. In our case, the p-value of this test turns out to be 0.55, thus not rejecting the null hypothesis of no sorting. We also conduct the density discontinuity test described in Cattaneo et al., 2018, which further confirms our earlier finding of no discontinuities in the distribution of the running variable (bottom panel of Figure A.7).

Additionally, we examine whether there are any differences in pre-determined characteristics at the cutoff point. The reasoning behind this check is to verify if the policy affected outcomes that were determined before it took effect, such as gender and tribe affiliation. Ideally, we should find null effects of the policy on these characteristics. The results of this exercise are presented in Figure A.8. Each graph represents the local average of the pre-determined characteristics by the running variable, age in 1980. The

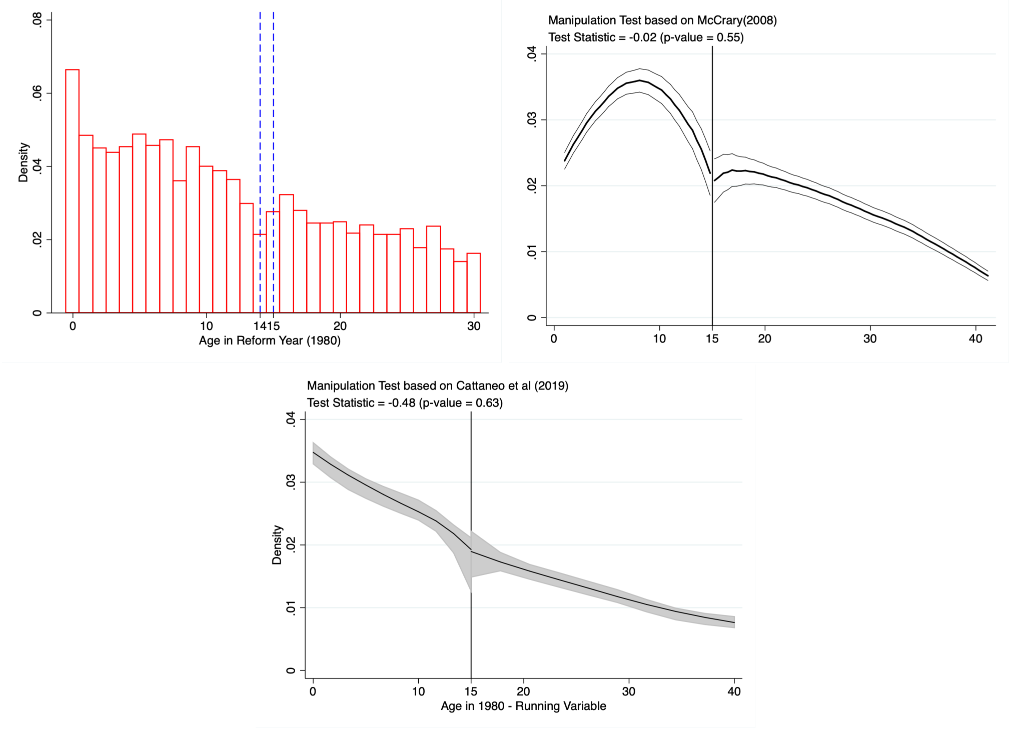


Figure A.7: **Distribution of running variable (age in 1980) and manipulation tests**

Notes: Based on Zimbabwe World Health Survey data. The upper left panel represents the distribution of the running variable (age in 1980). The upper right panel is the manipulation test based on McCrary, 2008. The lower panel is the manipulation test based on Cattaneo et al., 2018. The vertical line in each graph represents the cutoff ages.

graphs suggest no statistically significant jumps at the cutoff point for any of these covariates, showing that there is balance in the pre-determined covariates between the two groups.

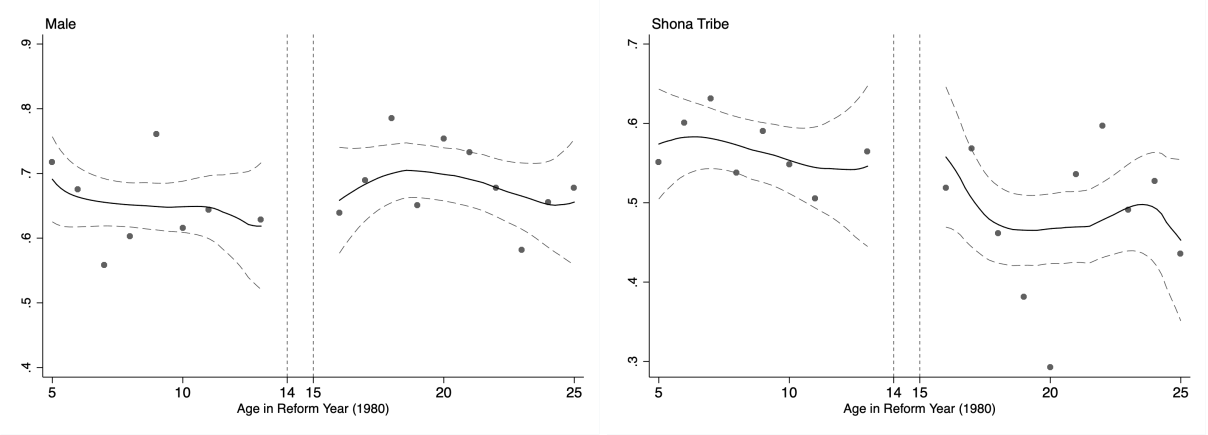


Figure A.8: **Falsification check – RD impact on predetermined outcomes.**

Notes: The figures plot predetermined covariates, male and tribe, in yearly bins against the running variable (age in 1980). Source: World Health Survey Zimbabwe.

One potential threat to our identification strategy is that the Zimbabwean government could have implemented other pro-social policies at the time of independence (in 1980), that could have an impact on our outcomes of interest. We suggest that this is unlikely

to be the case because the effects we identify are based on RD estimation around the age cutoff of 15 years, and to the best of our knowledge, there were no other policies specifically targeted based on the same age-based rule. Further, we conduct several falsification checks to add credibility to our findings.

# References

Cattaneo, M. D., Jansson, M., and Ma, X. (2018). Manipulation testing based on density discontinuity. *The Stata Journal*, 18(1):234–261.

Hahn, J., Todd, P., and Van Der Klaauw, W. (2001). Identification and estimation of treatment effects with a regression-discontinuity design. *Econometrica*, 69(1):201–209.

Lee, D. S. and Lemieux, T. (2010). Regression Discontinuity designs in economics. *Journal of Economic Literature*, 48(2):281–355.

McCrary, J. (2008). Manipulation of the running variable in the regression discontinuity design: A density test. *Journal of Econometrics*, 142(2):698–714.

Nhundu, T. J. (1989). The financing and provision of education in Zimbabwe: Towards greater equality? *Educational Review*, 41(3):243–253.

Nhundu, T. J. (1992). A decade of educational expansion in Zimbabwe: Causes, consequences, and policy Ccntradictions. *The Journal of Negro Education*, 61(1):78.
